# Expression and function of hypoxia inducible factor-1 alpha in human melanoma under non-hypoxic conditions

**DOI:** 10.1186/1476-4598-8-104

**Published:** 2009-11-17

**Authors:** Caroline N Mills, Sandeep S Joshi, Richard M Niles

**Affiliations:** 1 Department of Biochemistry and Microbiology, Joan C. Edwards School of Medicine, Marshall University, Huntington, WV 25755, USA; 2 Department of Pathology, University of Alabama, Birmingham, AL 35233, USA

## Abstract

**Background:**

Hypoxia inducible factor-1 alpha (HIF-1α) protein is rapidly degraded under normoxic conditions. When oxygen tensions fall HIF-1α protein stabilizes and transactivates genes involved in adaptation to hypoxic conditions. We have examined the normoxic expression of HIF-1α RNA and protein in normal human melanocytes and a series of human melanoma cell lines isolated from radial growth phase (RGP), vertical growth phase (VGP) and metastatic (MET) melanomas.

**Results:**

HIF-1α mRNA and protein was increased in RGP vs melanocytes, VGP vs RGP and MET vs VGP melanoma cell lines. We also detected expression of a HIF-1α mRNA splice variant that lacks part of the oxygen-dependent regulation domain in WM1366 and WM9 melanoma cells. Over-expression of HIF-1α and its splice variant in the RGP cell line SbCl2 resulted in a small increase in soft agar colony formation and a large increase in matrigel invasion relative to control transfected cells. Knockdown of HIF-1α expression by siRNA in the MET WM9 melanoma cell line resulted in a large decrease in both soft agar colony formation and matrigel invasion relative to cells treated with non-specific siRNA. There is a high level of ERK1/2 phosphorylation in WM9 cells, indicating an activated Ras-Raf-MEK-ERK1/2 MAPK pathway. Treatment of WM9 cells with 30 μM U0126 MEK inhibitor, decreased ERK1/2 phosphorylation and resulted in a decrease in HIF-1α expression. However, a 24 h treatment with 10 μM U0126 totally eliminated Erk1/2 phosphorylation, but did not change HIF-1alpha levels. Furthermore, siRNA knockdown of MEK siRNA did not change HIF-1alpha levels.

**Conclusion:**

We speculate that metabolic products of U0126 decrease HIF-1alpha expression through "off target" effects. Overall our data suggest that increased HIF-1α expression under normoxic conditions contributes to some of the malignant phenotypes exhibited by human melanoma cells. The expanded role of HIF-1α in melanoma biology increases its importance as a therapeutic target.

## Background

The incidence of melanoma is increasing more rapidly than any other tumor site. Melanoma accounts for 4% of all skin cancers, but for 79% of all skin cancer related deaths in the United States (Melanoma Research Foundation). Metastatic melanoma is highly resistant to both chemo- and radiotherapy [1]. Cutaneous melanoma arises from, melanocytes, presumably due to early childhood exposure of the skin to UV radiation. A predisposing factor for melanoma may be the melanocortin receptor. It has been found that individuals having a mutation that affects the function of the melanocortin receptor have an increased risk of developing cutaneous melanoma [2].

Hypoxia-inducible factor-1 (HIF-1) is a master regulator of O_2 _homeostasis in cells. It consists of a heterodimeric transcriptional complex of two proteins, HIF-1β and HIF-1α. HIF-1β is constitutively expressed whereas HIF-1α protein is stabilized only under hypoxic conditions, despite its continuous synthesis under normoxic conditions [3]. When O_2 _tension is normal, HIF-1⟨ is hydroxylated at specific proline residues by the enzyme prolyl hydroxylase-domain (PHD). This hydroxylation is required for the von Hippel Lindau (VHL) tumor suppressor protein to bind to HIF-1α leading to subsequent ubiquitination and proteasome-targeted degradation [4]. VHL binding is also enhanced by acetylation of lys^532 ^catalyzed by the acetyltransferase, ADP-ribosylation factor domain protein 1 (ARD1) [5]. Under hypoxic conditions, proline hydroxylation decreases thereby stabilizing HIF-1α, which in turn moves to the nucleus and transactivates various genes containing hypoxia response elements [6].

HIF-1α controls the expression of over 60 genes involved in many aspects of oncogenesis, including tumorigenesis [7,8] anti-apoptosis [9,10], and genetic instability [11]. HIFα has also been implicated in the malignant progression of several cancers including mammary gland, prostate, brain, and lung [12]. HIF-1α is the regulatory subunit of HIF-1. It is regulated at the protein level by both oxygen- dependent and independent pathways [6]. HIF-1α is highly expressed in early stage of mouse hepatocarcinogenesis independent of hypoxia [13]. The hypoxia independent increase in HIF-1α is thought to be activated by growth signaling pathways. A majority of human melanomas have constitutively active MAPK/extracellular signal-regulated kinase (ERK) due to BRAF or N-Ras mutations [14,15]. Activation of this pathway is correlated with the upregulation of HIF-1α mRNA in human melanoma [16,17]. However the biological significance of upregulated HIF-1α under normoxic conditions for initiation and progression of melanoma has not been elucidated.

In this study, we examined the normoxic expression and biological functions of HIF-1α in human melanoma. We found that both full length and a splice variant, HIF-1α785, are expressed in human melanoma cell lines while essentially undetectable in normal human melanocytes. Ectopic HIF-1α expression in a low expressing RGP cell line stimulated Matrigel invasion, while knockdown of HIF-1α in a high expressing MET cell line inhibited both soft agar colony formation and Matrigel invasion. Knockdown of MEK1/2 and loss of phosphorylated ERK1/2 did not decrease HIF-1α expression. U0126 MEK inhibitor at 10 μM eliminated ERK1/2 phosphorylation, but did not decrease HIF-1α expression.

## Results

### Expression of HIF-1α in human melanoma cells

**Figure 1:**
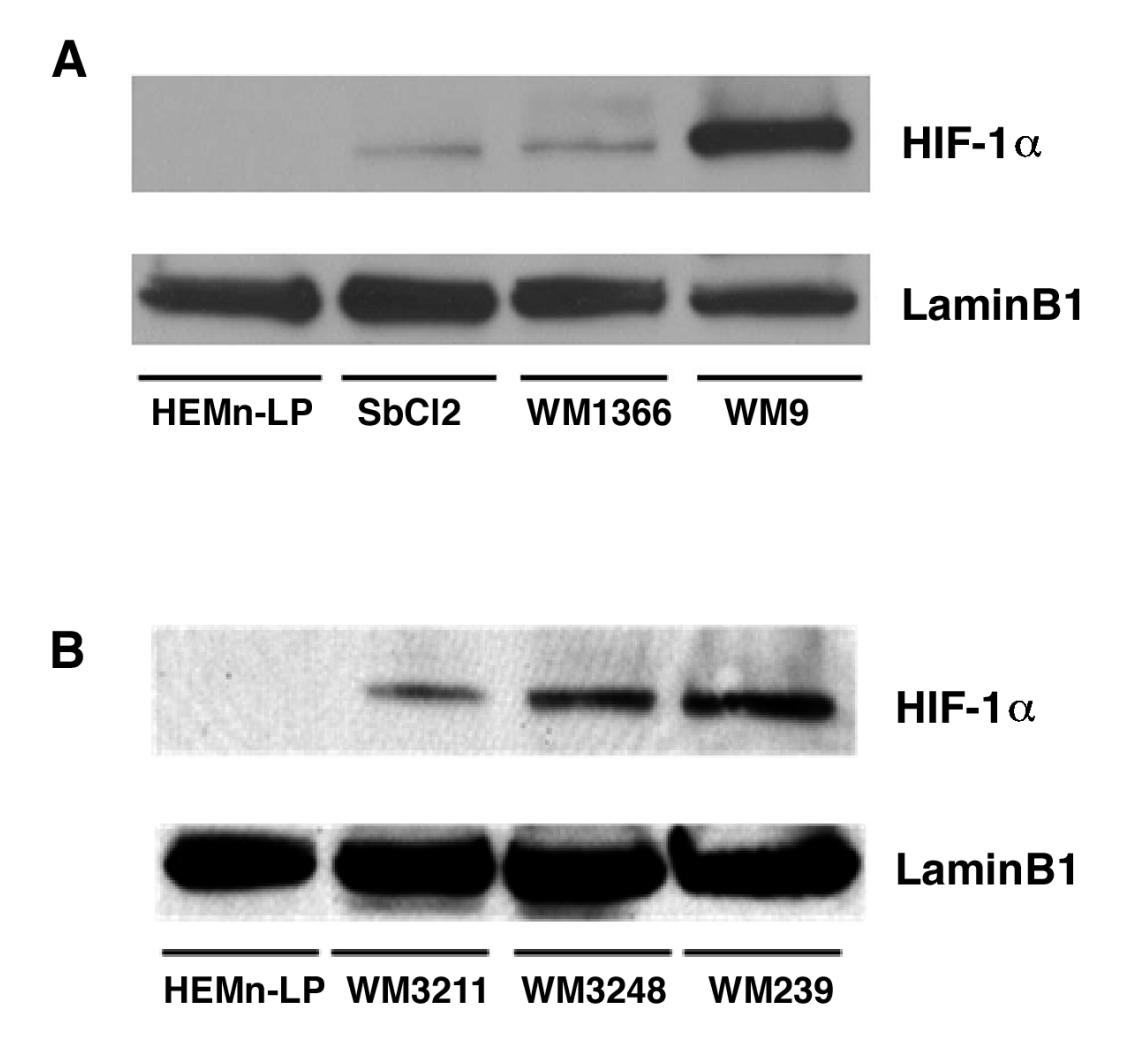
**Expression of HIF-1α protein in human melanocytes and melanoma cells**. Nuclear extracts (30 μg) from HEMn-LP (normal human melanocytes), SbCl2 (radial growth phase melanoma), WM1366 (vertical growth phase melanoma) and WM9 (metastatic melanoma) (A) and WM3211 (radial growth phase melanoma), WM3248 (vertical growth phase melanoma), WM239 (metastatic melanoma) (B) cell lines were analyzed by western blotting using a monoclonal anti-HIF-1α antibody (1 μg/ml). LaminB1 was used as a loading control. This blot is representative of at least 3 different experiments, all of which gave similar results.

In addition to the well known pathway of HIF-1alpha protein stabilization under hpoxic conditions, it has been established that many oncoproteins and growth factor signaling pathways up-regulate HIF-1α expression under normoxic conditions [18,19]. However, there are few investigations into the normoxic expression of HIF-1α in human melanoma and its role in the malignant progression of this disease. Here, we show that in human melanoma cells, the oxygen-labile HIF-1α protein as well as its mRNA is expressed endogenously under normoxic conditions. Figure 1A shows that HIF-1α protein is highly expressed in WM9 cells relative to normal human melanocyte (HEMn-LP), but radial growth phase (SbCl2), and vertical growth phase (WM1366) also express a higher amount of HIF-1α protein relative to human melanocytes. Similar results are seen in second set of RGP, VGP and MET melanoma cell lines (Fig. 1B). HIF-1α was detected as 120 kD protein in nuclear extracts while no protein was detected in cytoplasmic extracts (data not shown).

**Figure 2:**
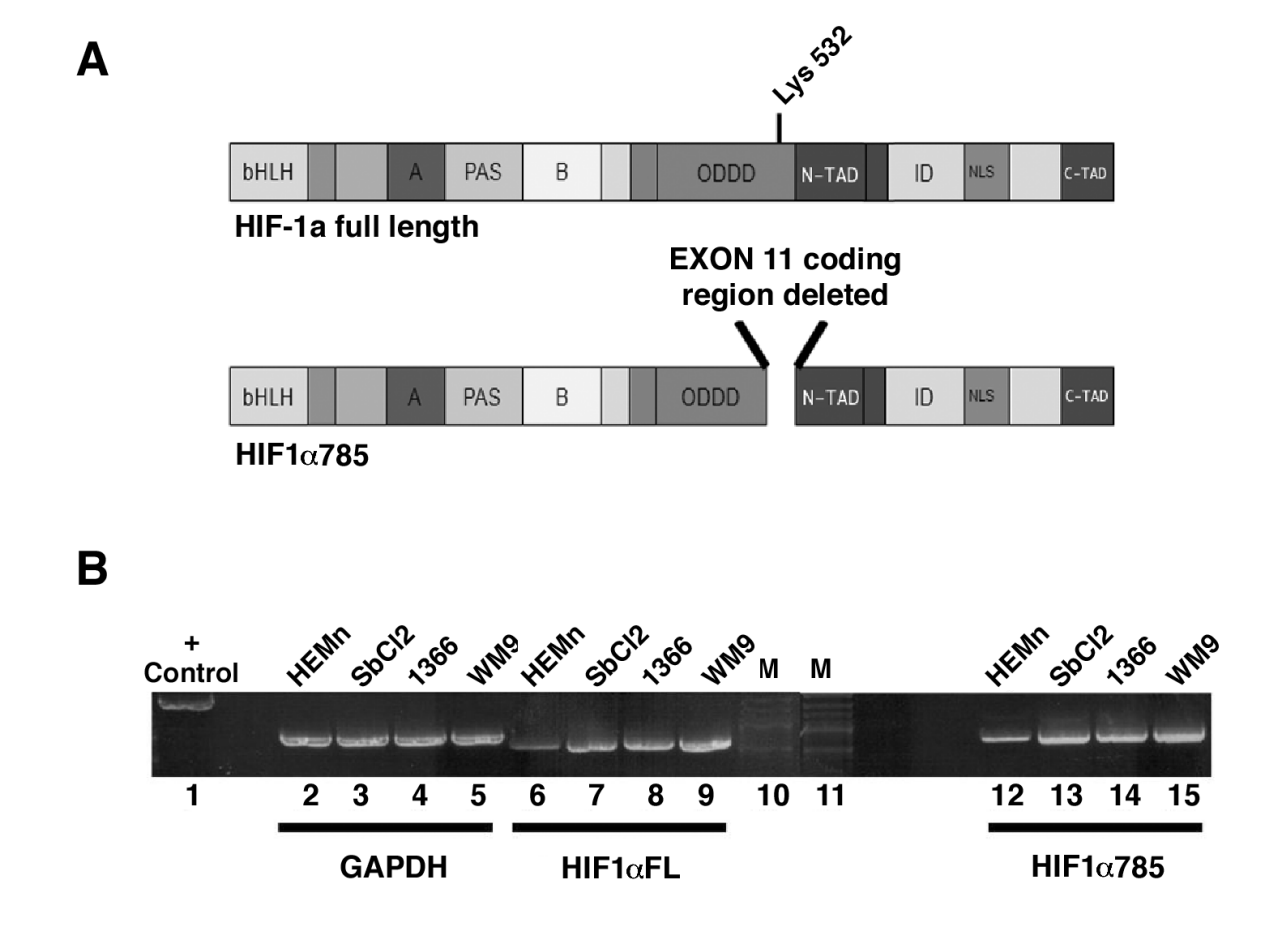
**Expression of HIF-1αFL & HIF-α785 mRNA in human melanoma cells**. (A) Schematic representation of the functional domains of both HIF-1α full length and HIF-1α785. Both HIF-1αFL and 785 have various domains in common such as basic helix loop helix (bHLH), Per/Arnt/Sim (PAS), Oxygen Dependent Degradation Domain (ODDD), N-terminal transactivation domain N-TAD, inhibitory domain (ID), nuclear localization signal (NLS) and C-terminal transactivation domain C-TAD. Upon loss of exon 11 in HIF-1α785, part of the ODDD is deleted. This missing region contains the important lysine ^532 ^residue which is acetylated by ARD1 leading to increased stable interaction of HIF-1α with the von Hippel Lindau tumor suppressor. This interaction directs HIF-1α to the ubiquitin-proteasome pathway for degradation under normoxic conditions [5]. (B) Total RNA was extracted from HEMn-LP, SbCl2, WM1366, and WM9. RNA was reverse transcribed using the Advantage RT-for PCR kit^®^. Five μL of the resulting cDNA was used in the PCR reaction as described in the Advantage cDNA kit^® ^manual. Primers (see Methods for sequence) for HIF-1α and HIF-1α785 were designed to specifically amplify each variant with no cross-amplification. Primers for the housekeeping control gene GAPDH were included in the Advantage cDNA kit^®^. Control primers amplifying a fragment of the control plasmid included in the Advantage cDNA kit^® ^were used to ensure optimal PCR conditions.

Hypoxic stabilization of HIF-1α occurs at the protein level [3]. Since HIF-1α protein was increased in human melanoma cells under normoxic conditions, we determined whether this increase might be due to increased HIF-1α mRNA levels. Initially we used semi-quantitative RT-PCR to assess expression of HIF-1α full length (FL) and a splice variant HIF-1α785 that is missing the acetylation site lys^532 ^due to lack of exon 11 (Fig. 2A). This splice variant encodes HIF-1α protein that has been reported to be stable under normoxic conditions [17,20]. Primers were designed so that full length HIF-1α would exclude HIF-1α785 by targeting exon 11, which is absent in HIF-1α785. Primers for HIF-1α785 excluded HIF-1α by targeting the exon 10:12 boundary only present in HIF-1α785. Fig. 2B shows that human melanoma cell lines express both full-length and the 785 splice variant HIF-1α mRNA at a level that appeared to be higher than normal human melanocytes.

**Figure 3:**
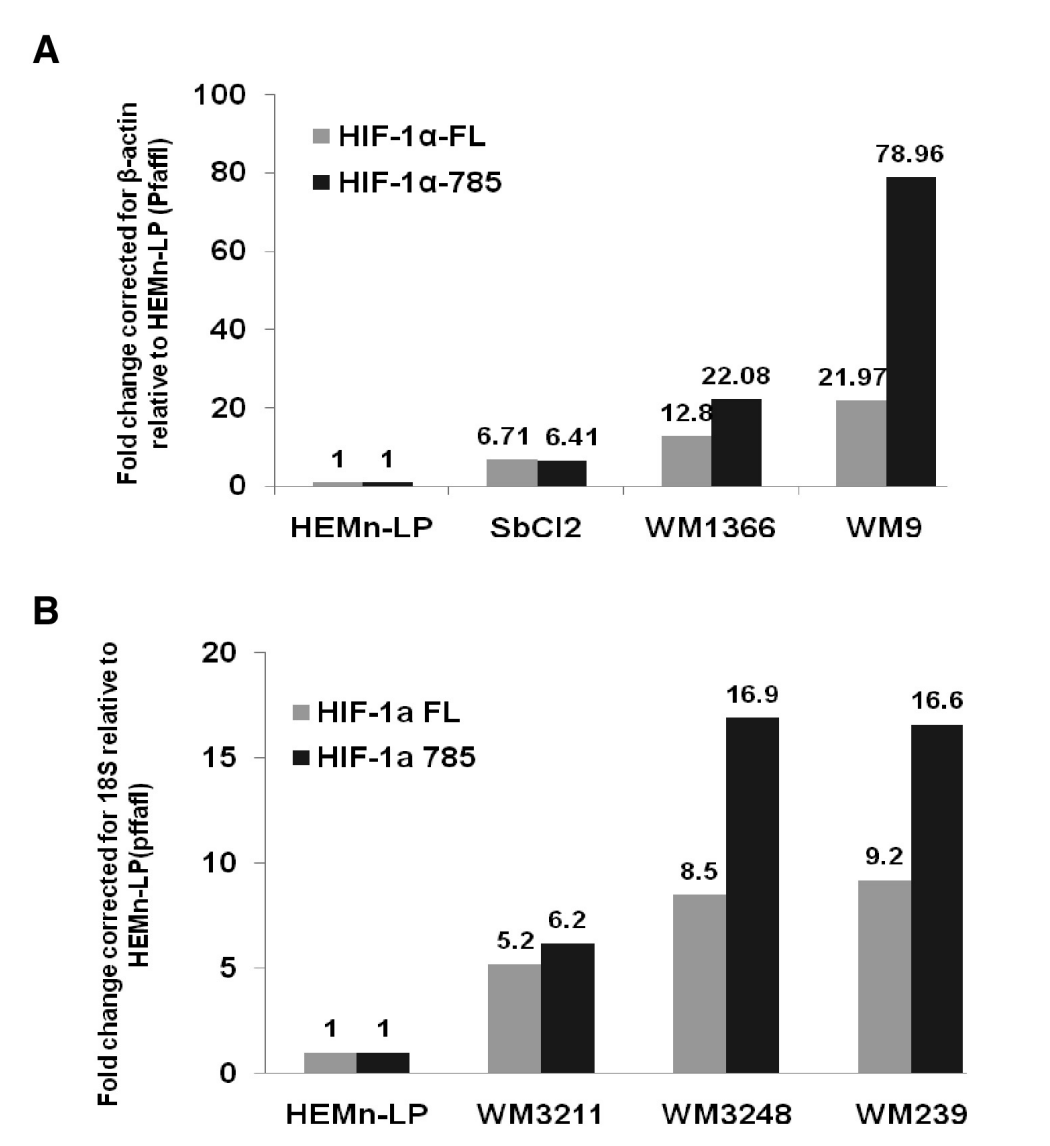
**Quantitative analysis of HIF-1αFL & HIF-1α785 mRNA expression in human melanoma cells**. Total RNA was extracted from HEMn-LP, SbCl2, WM1366, and WM9 cells at 72 h (A) after seeding. RNA was then converted to cDNA using the High Capacity cDNA Archive Kit (ABI). Real-Time PCR analysis was performed using TaqMan probes directed at HIF-1αFL or HIF-1α-785 as well as β-actin. The reactions were performed under conditions specified in the ABI TaqMan Gene Quantification assay protocol. Data was corrected for efficiency and loading using the Pfaffl method. Data is expressed as fold change, corrected for β-actin, relative to HeMn-LP. (B) Real-time PCR analysis was performed using Taqman probes directed at HIF-1αFL or HIF-1α785 as well as 18S using RNA extracted from HEMn-LP, WM3211, WM3248, and WM239 cells. Data is representative of at least 2 separate experiments.

These findings were verified by qRT-PCR measurement of full-length and HIF-1α785 mRNA levels (Fig. 3). All melanoma cell lines had increased expression of HIF-1α mRNA relative to normal human melanocytes. In addition VGP and MET cell lines expressed more of the 785 HIF-1α mRNA than full length HIF-1α mRNA. Overall the WM9 metastatic melanoma expressed the highest amount of 785 HIF-1α mRNA (~79× higher than normal human melanocytes).

### HIFαFL and HIF-1α785 gain-of-function in radial growth phase SbCl2 cells

**Figure 4:**
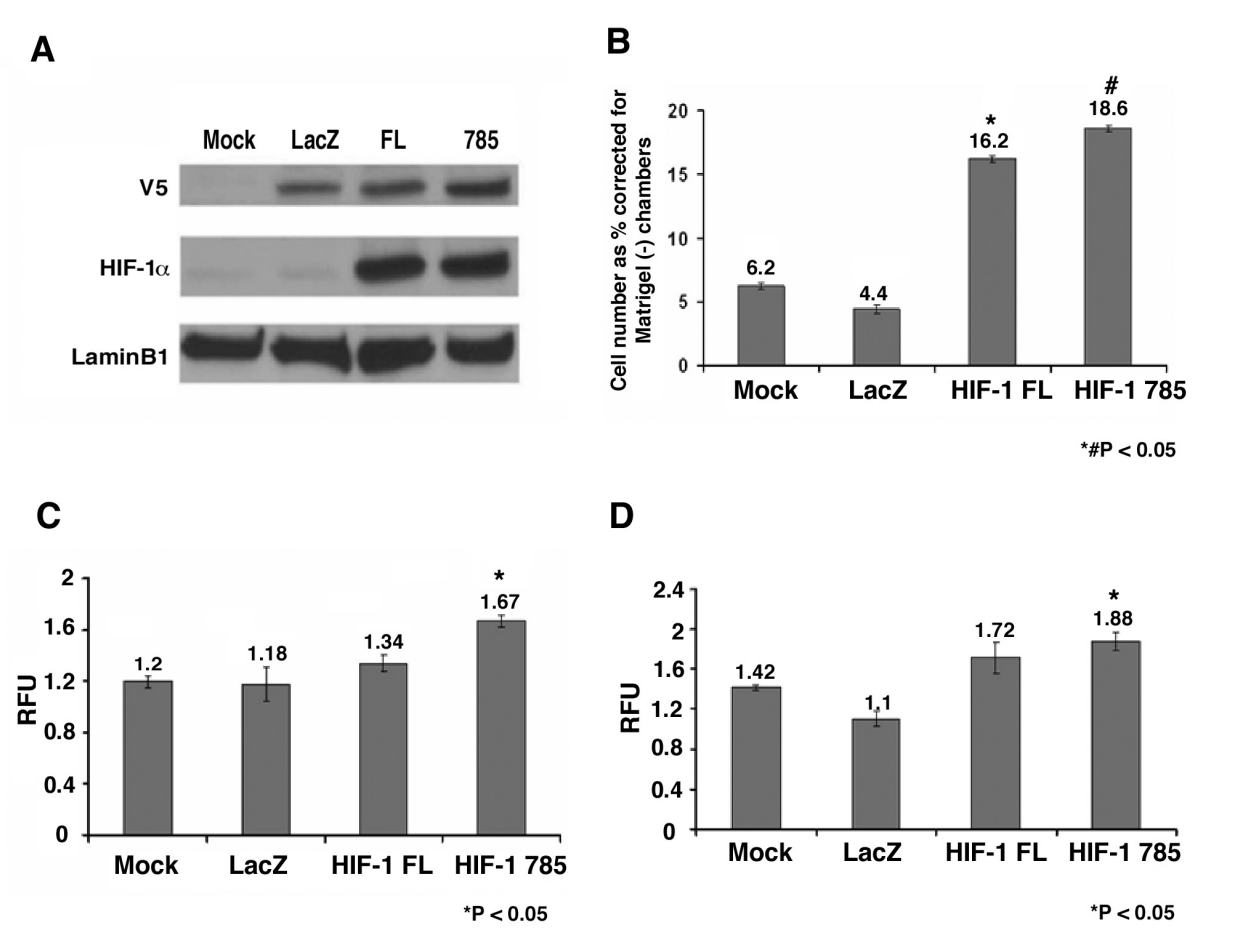
**Effect of HIF-1αFL and HIF-1α785 overexpression in radial growth phase SbCl2 cells on anchorage-independent growth and invasion**. SbCl2 cells were transiently transfected at 80% confluence with either mock (no plasmid DNA), pLenti-V5-Lacz, pLenti-V5-D-TOPO-HIF-1αFL or pLenti-V5-D-TOPO-HIF-1α785 using FuGene 6 transfection reagent. (A) After 48 h nuclear protein was extracted and over-expression was confirmed by western blot using HRP-conjugated anti-V5 antibody and mouse monoclonal anti-HIF-1α antibody (1 μg/ml). LaminB1 was used as loading control. (B) SbCl2 cells 24 h post transfection with either mock (no plasmid DNA), Lacz, HIF-1αFL or HIF-1α785 were subjected to Matrigel invasion assay as described in "Material and Methods". Results are expressed as percent invasive cells corrected for invasion level of cells seeded in Matrigel (-) chambers. (C&D). SbCl2 cells transfected either with reagents alone (mock), Lacz, HIF-1αFL or HIF-1α785 were subjected to CytoSelect 96 well Cell Transformation Assay^® ^(Cell Biolabs, Inc.) as described in "Material and Methods" for 4 (C) and 5 days (D). Results are expressed as Relative Fluorescent Units. Data is expressed as the mean ± SEM of triplicate values. ANOVA with TUKEY for multiple pairwise comparison was used to analyze the data and all P values ≤ 0.05 are relative to Mock and Lacz overexpressing cells. The entire experiment was repeated two additional times with similar results.

The level of HIF-1α protein is low in the radial growth phase SbCl2 cells relative to VGP or MET cell lines. We determined the effect of HIF-1αFL or HIF-1α785 overexpression on SbCl2 anchorage-independent growth and Matrigel invasion. HIF-1αFL and HIF-1α785 were cloned into the pLenti-V5-D-TOPO vector and transiently overexpressed in SbCl2 cells (Fig. 4A). HIF-1α785 overexpression resulted in a small, but statistically significant increase in anchorage-independent growth, relative to mock or lacz transfected cells (Fig. 4C and 4D). In contrast overexpression of both HIF-1αFL and HIF-α785 in SbCl2 resulted in a large and significant 3-fold increase in Matrigel invasion relative to mock or Lacz transfected cells (Fig. 4B).

### HIF-1α loss-of-function in human metastatic melanoma WM9 cells

**Figure 5:**
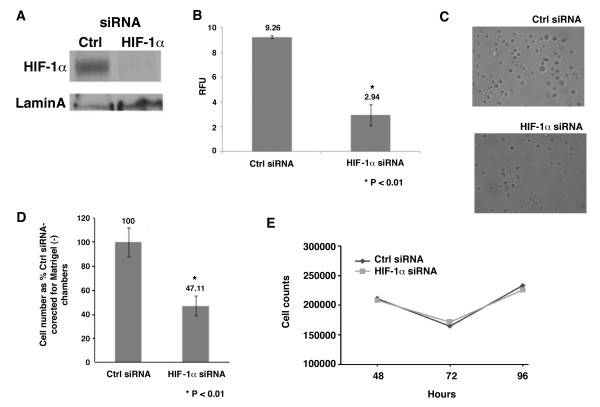
**The effect of loss of HIF-1α expression in human metastatic melanoma WM9 cells on anchorage-independent growth and invasion**. (A) WM9 cells were treated with either 100 nM HIF-1α siRNA or 100 nM control non-targeting siRNA (Dharmacon, Inc.) using the RNAifect^® ^transfection reagent (Qiagen, Inc.). Knock down of HIF-1α was confirmed by western blot at 72 h post transfection. (B) At 48 h post transfection Ctrl siRNA or HIF-1α siRNA treated WM9 cells, were assayed for anchorage independent growth using the CytoSelect 96 well Cell Transformation Assay^® ^(Cell Biolabs, Inc.). Briefly, the cells were seeded at 8.0 × 10^3 ^cells/well into a 0.4% agar layer poured over a 0.6% agar layer of a 96 well plate. Wells lacking cells served as a blank control. On day 5 after seeding, agar layers were solubilized, cells were lysed, and nucleic acid stained with CyQuant dye. Fluorescence intensity in each well was determined by a plate reader set at 485/520 nm. Results are expressed as Relative Fluorescent Units. (C) Photomicrograph of a representative field of colonies form in the Cell Transformation Assay by control siRNA transfected cells (left) vs. HIF-1α siRNA transfected cells. (D) Matrigel invasion assay. At 48 h post transfection, the Ctrl siRNA or HIF-1α siRNA transfected WM9 cells were seeded into 6-well Matrigel (+) chambers, and as a control, 6-well Matrigel (-) chambers (BD Biosciences) at 7.0 × 10^4 ^cells per well. The method and counting of invading cells was done as described in "Experimental Procedures". Results are expressed as % invasion of HIF-1α siRNA treated-WM9 cells relative to invasion by Control siRNA treated-WM9 cells corrected for invasion by similarly treated cells seeded in Matrigel (-) chambers. (E) Cell viability assay was done in WM9 cells transfected with control siRNA or HIF-1α siRNA at 48, 72 and 96 h following transfection by the trypan blue exclusion method. Data is expressed as the mean ± SEM of triplicate samples. The entire experiment was repeated twice with similar results. Student paired *t *test was used to analyse the data and all P values ≤ 0.01 or 0.05 are relative to control siRNA treated cells.

HIF-1α protein is highly expressed under normoxic conditions in the WM9 human metastatic melanoma cell line. To determine whether HIF-1α could be contributing to the malignant characteristics of these cells, we knocked down its expression and examine how this affected anchorage independent growth and Matrigel invasion. WM9 cells were treated with 100 nM siRNA targeting HIF-1α (Dharmacon) which consistently decreased its expression by ~75-85% (Fig. 5A). Colony formation after 5 days in soft agarose was inhibited by 70% in HIF-1α-siRNA transfected WM9 cells in comparison to cells transfected with control siRNA (Fig. 5B). A photo (Fig. 5C) of the colonies formed at this time point in control vs. HIF-1α transfected cells verifies this decrease in soft agar colony formation. Matrigel invasion was also significantly decreased in HIF-1α-siRNA transfected WM9 cells compared to control siRNA transfected WM9 cells (Fig. 5D). Measurement of cell viability in the Matrigel chambers shows no difference between control vs. HIF-1α siRNA transfected cells (Fig. 5E). These knock down studies suggest that increased non-hypoxic expression of HIF-1α plays an important role in key malignant properties exhibited by these human melanoma cells.

### Regulation of HIF-1α expression in human melanoma by the ERK1/2 MAPK pathway

**Figure 6:**
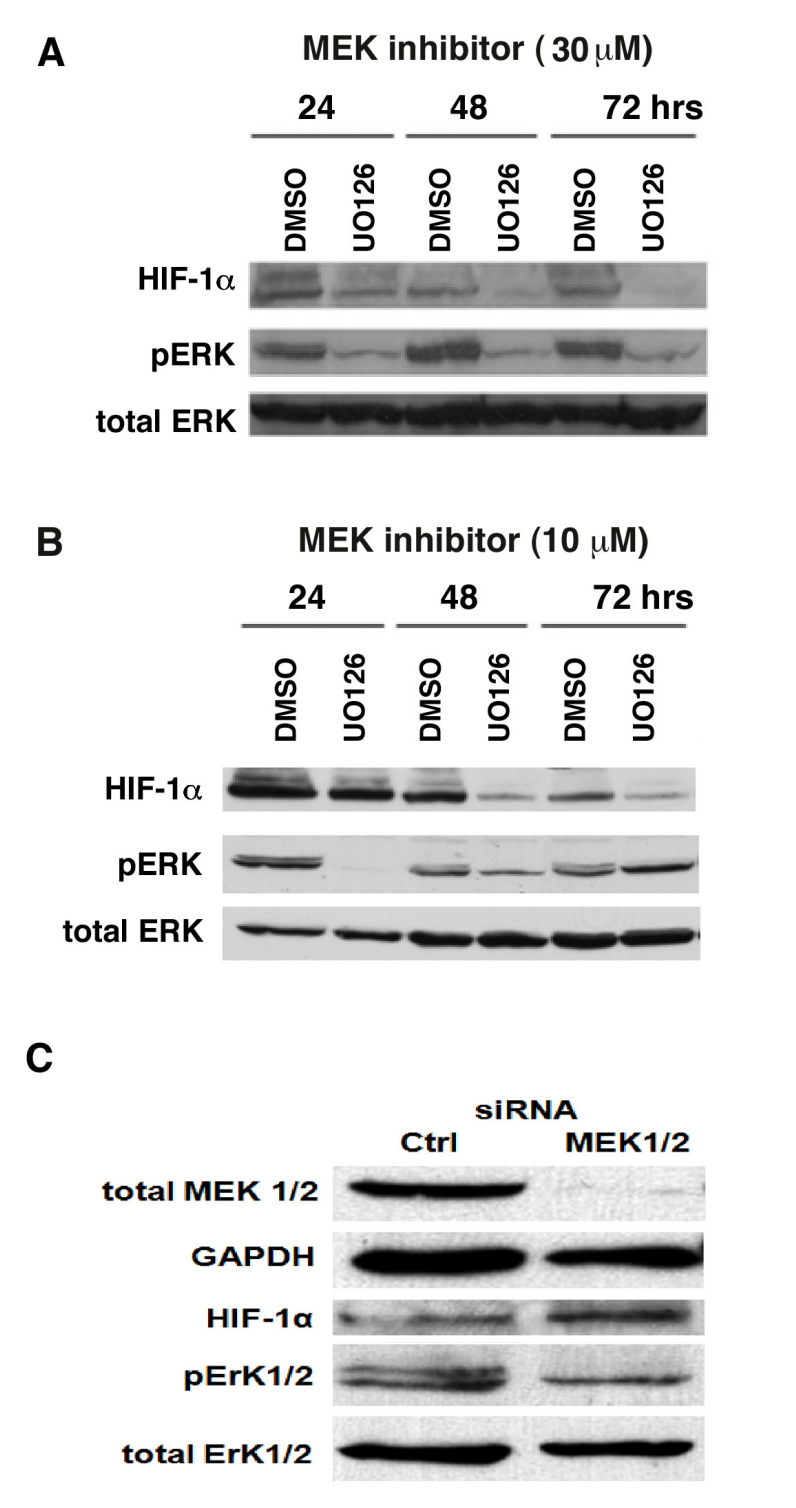
**Effect of ERK1/2 MAPK inhibition on HIF-1α expression in human melanoma cells**. WM9 cells were treated with either 30 (A) or 10 (B) μmol/L U0126 a MEK1/2-specific inhibitor or vehicle (DMSO). Inhibition of ERK phosphorylation and HIF-1α expression was determined by western blotting at 24, 48 and 72 h after treatment using both total ERK, phospho-specific ERK and HIF-1α antibodies respectively. C. WM9 cells were also treated with either 100 nM MEK1&2 siRNA or 100 nM control non-targeting siRNA (Dharmacon, Inc.) using the RNAifect^® ^transfection reagent (Qiagen, Inc.). Knock down of MEK1&2 was confirmed by western blot at 72 h post transfection. Inhibition of ERK phosphorylation and HIF-1α expression was examined by western blot at the same time point in nuclear extracts by using both total ERK, phospho-specific ERK, and HIF-1α antibodies. The entire experiment was repeated two additional times with similar results.

Hypoxia independent expression of HIF-1α is thought to be regulated by growth signaling pathways [18,19] and the majority of melanomas have constitutively active ERK1/2 MAPK pathway due to BRAF or N-Ras mutations [14,15]. Therefore, we determined whether HIF-1α expression in human metastatic melanoma WM9 cells was dependent on activation of ERK1/2 MAPK signaling. These cells have an active ERK1/2 MAPK pathway as evidenced by the high phosphorylation of ERK (Fig. 6A). Treatment of WM9 cells with 30 μM U0126, a selective U0126 MEK inhibitor, decreased ERK1/2 phosphorylation and led to a time-dependent decrease in HIF-1α protein expression (Fig. 6A). Although 30 μM U0126 has been used in published studies to selectively inhibit MEK [16,21], the original paper describing this inhibitor [22] used much lower concentrations to achieve high selectivity. Therefore we repeated this experiment using 10 μM U0126 (Fig. 6B). At 24 h of treatment, 10 μM U0126 completely suppressed the phosphorylation of ERK1/2, yet there was minimal change in the level of HIF-1α relative to control cells. With further time of inhibitor treatment, phosphorylation of ERK was not totally suppressed, but HIF-1α levels decreased. We also used siRNA specifically targeting MEK1 and 2 in WM9 cells to inhibit ERK1/2 phosphorylation. Treatment of WM9 cells with siRNA targeting MEK1 and 2 consistently decreased its expression by greater than 90% and also decreased ERK1/2 phosphorylation. However, knockdown of MEK1 and 2 did not decrease the normoxic expression of HIF-1α protein in human metastatic melanoma WM9 cells (Fig. 6C).

## Discussion

Melanocytes - the cells responsible for producing the skin-coloring pigment, melanin, are the point of origin for melanoma. Melanoma, if diagnosed and treated early, has a high cure rate [23]. If the melanoma progresses, it can metastasize regionally to lymph nodes, and then to distant organs such as the lungs, and the brain [24]. Metastatic melanoma is very difficult to treat and has a high mortality rate. Several studies have confirmed that HIF-1α is a survival factor, as well as a key regulator of metastasis in various cancers [25,26].

HIF-1α regulates the adaptive responses to O_2 _tensions at cellular levels. It controls the expression of many genes involved in different aspects of tumor biology, including angiogenesis, cell survival, invasion [3], tumor growth [7,8], anti-apoptosis [9,10], and genetic instability [11]. HIF-1α is rapidly degraded under normoxic condition and is stablilized under hypoxic condition [3]. We found that HIF-1α protein and RNA is expressed under normoxic conditions in several human melanoma cell lines and that the levels of HIF-1α expression correlates with the stage of cancer from which the melanoma cell line was established. In contrast, HIF-1α protein was undetectable in normal human melanocytes. Normoxic expression of HIF-1α has been found in a number of cancer cell types [13,17,27]. Activation of the ERK1/2 MAPK [16] and phosphotidylinositol 3-kinase (PI3K) [18] pathways has been implicated in stimulating normoxic expression of HIF-1α.

The epidermis of the skin is a partial hypoxic environment [28]. Evidence has been provided that this partial hypoxia contributes to melanomagenesis [29,30]. Therefore, one possibility is that the increase in HIF-1α that we observed in melanoma ceils is due to the hypoxic adoptive response maintained by the cells in culture. However, hypoxia stabilizes the HIF-1α protein and does not increase the level of HIF-1α mRNA. We found that the increased HIF-1α protein in the melanoma cells was correlated with an increase in HIF-1α mRNA. Also, the normal human melanocytes used in our study came from the partial hypoxic environment of the skin and yet in culture, they do not express detectable levels of HIF-1α protein. Therefore, we think it more likely that the increased HIF-1α mRNA and protein is due to an inappropriately activated signaling pathway.

We also found that an mRNA splice variant, HIF-1α785, was expressed at higher levels than full length HIF-1α mRNA in the VGP and metastatic human melanoma cell lines. HIF-1α785 is missing the acetylation site lys^532 ^due to lack of exon 11 and is thought to be more stable under normoxic conditions in comparison to full length HIF-1α [17,20]. In several non-melanoma cell lines it was found that phorbol ester stimulated the expression of HIF-1α785 mRNA under normoxic conditions via a redox-dependent ERK1/2 MAPK pathway [17]. How this alternatively spliced isoform of HIF-1α mRNA is increased in melanoma is currently under investigation.

We examined the biological consequences of HIF-1α FL and 785 splice variant gain of function and HIF-α FL loss of function in selected human melanoma cells. The SbCl2 radial growth phase melanoma cells have low levels of HIF-1α protein expression and a limited capacity to form colonies in soft agar and to invade through Matrigel. This cell line was chosen to determine the biological effects of HIF-1α overexpression. Transient ectopic expression of FL HIF-1α did not result in a statistically significant increase in SbCl2 colony formation in soft agar. However, transient overexpression of HIF-1α785 resulted in a small, but statistically significant increase in soft agar colony formation. The effect on anchorage-independent growth may be limited by the transient nature of the overexpression of HIF-1. Overexpression of either HIF-1αFL or 785 led to a large and statistically significant increase in the ability of SbCl2 cells to invade Matrigel. There have been a few reports on the effect of HIF-1α overexpression on the in vitro biologic properties of cancer cells. Hypoxia-induced or exogenous overexpression of HIF-1α increased *in vitro *invasion by human colon adenocarcinoma cells [31], while stable normoxic overexpression of HIF-1α promoted anchorage-independent growth in melanocytes having an activated AKT signaling pathway [30]. SbCl2 cells overexpressing HIF-1α785 showed a somewhat greater increase in soft agar colony formation as well as invasion ability compared to full length HIF-1α. Whether this difference is due to a longer half-life for the splice variant protein relative to the full length protein is currently under investigation.

HIF-1α loss of function experiments were carried out in the WM9 metastatic melanoma cell line. This cell line was chosen due to its high level of normoxic expression of HIF-1α. The WM9 is an aggressive metastatic melanoma cell line that has a high level of anchorage-independent growth and Matrigel invasion ability. We found a significant decrease in both anchorage-independent growth and Matrigel invasion upon silencing of normoxic expression of HIF-1α by siRNA treatment. These decreases were not due to a loss of cell viability as has been reported for knockdown of HIF-1α under hypoxic conditions [16]. This decrease in invasion might be due to decreased expression of HIF-1α regulated genes involved in invasion such as matrix metalloproteinase 2 (MMP2), urokinase plasminogen activator receptor (uPAR), and cathepsin D [31,32]. Loss of anchorage-independent growth in HIF-1α silenced cells may be due to ERK/MAPK, PI3K/Akt and HIF-1 pathway interactions. The PI3K/Akt pathway is one of the most critical pathways involved in anchorage-independent growth [33].

Hypoxia independent expression of HIF-1α is thought to be regulated by growth signaling pathways [6]. The majority of melanomas have constitutively active ERK1/2 MAPK pathway due to BRAF or N-Ras mutations [14,15]. In particular, human metastatic melanoma WM9 cells have a constitutively active ERK1/2 MAPK pathway most likely due to the V600E BRAF mutation found in these cells. Treatment of these cells with 30 μM of the selective MEK inhibitor, U0126, decreased ERK1/2 phosphorylation and also resulted in a time dependent decrease in HIF-1α protein expression.

This 30 μM concentration chosen for our initial studies was based on two other published papers that used this amount of U0126 to demonstrate the involvement of the ERK1/2 MAPK pathway in the regulation of HIF-1α [16,21]. In the original report describing U0126, it was stated that the Ki for intracellular inhibition of ERK phosphorylation in COS-7 cells was 0.1 μM [22]. Thus the concentration used in our study and others [16,21] is 300 times higher than the Ki. Therefore we repeated the MEK inhibition studies using 10 μM U0126. In contrast to 30 μM U0126, the lower concentration completely eliminated ERK1/2 phosphorylation after a 24 h incubation with WM9 human metastatic melanoma cells. Despite this inhibition of ERK1/2 phosphorylation, there was no change in the expression of HIF-1α protein. At the later time point of 48 and 72 h of inhibitor treatment the phosphorylation of ERK1/2 was not completely suppressed and the level of HIF-1α protein was decreased. We also inactivated ERK1/2 signaling by knocking down the expression of MEK. We needed to use siRNA against both MEK1 and MEK2 in order to obtain a > 90% decrease in expression of these enzymes. Although this knockdown inhibited ERK phosphorylation, there was no decrease in HIF-1α protein expression at any time point assayed (up to 6 days). In the course of analyzing these data, we were informed by Promega, the manufacturer of U0126, that the compound is unstable in tissue culture media and produces metabolites that have poor MEK inhibitory activity. Considering the sum of our data, we hypothesize that the metabolites of U0126 are responsible for the decrease in HIF-1α protein levels. This would explain the lack of change in HIF-1α protein in cells treated for 24 h with 10 μM U0126 despite complete inhibition of ERK1/2 phosphorylation and the fact that siRNA knockdown of MEK resulting in decrease ERK1/2 phosphorylation also did not result in a decrease in HIF-1α protein levels.

Analysis of BRAF and NRAS mutations in our human melanoma cell lines (Table 1) shows that WM3211 cells do not have the common activating mutations in these genes, yet these cells express increased amounts of HIF-1α protein and mRNA under normoxic conditions. Overall our data suggest normoxic expression of HIF-1α is not regulated by the ERK1/2 MAPK pathway, *at least in the WM9 human metastatic melanoma cell line*. The hypoxia independent expression in melanoma cells, like other cancers, might be regulated by phosphotidylinositol 3-kinase (PI3K) [13,18], NFkB [34] or JAK/STAT [35] pathways.

**Table 1:** Genotyping data of melanoma cell lines

Mutation Cell lines	NRAS	WT/WT	V600E BRAF	V600D BRAF
Sbcl2	++			

WM3211		++		

WM1366	++			

WM3248			++	

WM9			++	

WM239				++

The N-RAS or BRAF mutational status of melanoma cell lines RGP (Sbcl2, WM3211), VGP (WM1366, WM3248), MET (WM9, WM239).

## Conclusion

In conclusion, HIF-1α is overexpressed, in melanoma cell lines under normoxic conditions in a manner that correlates with the aggressiveness of the tumor from which the cell line was established. We also show that the novel splice variant HIF-1α785, which is missing part of the oxygen regulation domain is overexpressed in these melanoma cell lines. Manipulation of HIF-1α expression in several of our melanoma cell lines suggests that this transcription factors regulates, in part, anchorage-independent growth and Matrigel invasion. Our results suggest that development of new therapeutic agents that inhibit HIF-1α function may be of use in the treatment of human melanoma regardless of the hypoxic condition of the tumor.

## Materials and methods

### Cell lines and cell culture conditions

SbCl2, WM3211 (RGP), WM1366, WM3248 (VGP), and WM9, WM239 (Metastatic melanoma) cells were a generous gift from Meenhard Herlyn's lab at the Wistar Institute (University of Pennsylvania). All cells were grown in a humidified incubator with 5% CO_2 _and 95% air at 37°C. The SbCl2 cells were cultured in MCDB153 media (Invitrogen, Carlesbad CA), supplemented with 2% fetal bovine serum, 5 μg/ml insulin (Sigma Chemical, St. Louis, MO), 1.68 mM CaCl_2_, 100 units/mL penicillin streptomycin solution (Invitrogen Corp., Carlsbad CA). WM3211 cells were cultured similar to Sbcl2 media except for 5% fetal bovine serum and no CaCl2. WM1366, WM3248, WM9 and WM239 cells were cultured in RPMI medium (Invitrogen Corp., Carlsbad CA) supplemented with 10% fetal bovine serum and 100 units/mL penicillin streptomycin solution. Normal human melanocytes (HEMn-LP) were derived from human foreskin (Cascade Biologics, Portland, OR) and maintained in Medium 254 supplemented with 50 mL HMGS (Cascade Biologics, Portland, OR) and 1 mL PSA solution (Cascade). Maintaining all cell lines in RPMI media for 48 h did not change their level of HIF-1α expression levels

### Western Blot analysis

Nuclear extracts from each cell line were isolated using the NePER kit^® ^(Pierce, Rockford, IL) according to the manufacturers protocol. Protein concentration was determined using the BCA protein assay reagents from Pierce as per the manufacturer's instructions. Proteins were separated by SDS-PAGE and transferred to nitrocellulose membranes using the BioRAD MiniProtean3^® ^system. Equal loading was also determined by Ponceau staining of the nitrocellulose membranes. Blots were blocked using ChemiBlocker reagent (Chemicon, Temecula, CA) for 1 hr at room temperature and probed overnight at 4°C with anti-HIF-1α mouse monoclonal antibody at 1 μg/mL (R&D Systems, Minneapolis, MN), 1:1000 phospho-p44/42 MAPK (Cell Signaling Tech Inc, Danvers, MA), p44/42 MAPK (Cell signaling Tech Inc, Danvers, MA), total MEK1/2 (Cell Signaling Tech Inc, Danvers, MA), 1:2000 V5-HRP (Invitrogen, Carlsbad, CA), 1 μg/mL Lamin B1 or Lamin A (Abcam, Cambridge, MA). Monoclonal mouse secondary IgG antibody (GE Healthcare, Piscataway, NJ) or rabbit secondary IgG antibody (Cell signaling) conjugated with HRP was applied after two 1× TBS + 0.05% Tween (TBS-T) washes. Blots were incubated with secondary antibody at 1:3000 for 1 h at room temperature and subsequently washed 2× with 1× TBS-T. A final 5 min wash with TBS (no Tween) was performed just prior to incubating the blot with ECL reagent (GE Healthcare, Piscataway, NJ). Blots were then autoradiographed and the density of immunoreactive bands determined by a BioRad imaging system after correction by an internal protein standard such nuclear lamin.

### RNA isolation and RT- PCR

Total RNA was extracted using Tri-Reagent (Sigma Chemical Co., St. Louis, MO). The purified RNA samples were dissolved in RNase-free water and quantified by Nanodrop spectrophotometer (NanoDrop Technology, Inc., Wimington, DE). Each RNA sample had an A260/A280 ratio of 1.8 or above. The quality of RNA was determined on the Agilent 2100 Bioanalyzer, using the RNA 6000 Nano Assay kit (Agilent Tehcnologies, Wilmington, DE) and reverse transcribed using the RT-for-PCR kit (Clontech, Palo Alto, CA) as per manufacturer's instructions. Primers were designed to either amplify only HIF-1α or HIF-1α785 exclusively. HIF-1α primers excluded HIF-1α785 by targeting exon 11, which is absent in HIF-1α785. HIF-1α785 primers were designed to exclude HIF-1α by targeting the exon 10:12 boundary only present in HIF-1α785. Sequence of the HIF-1α forward primers were: 5'-AAAGTTCACCTGAGCCTAAT-3', and reverse 5'-TAAGAAAAAGCTCAGTTAAC-3'. The sequence of HIF-1α785 forward primers were 5'-AAAGTTCACCTGAGGACAC-3', and reverse 5'-TAAGAAAAAGCTCAGTTAAC-3'. Primers for the housekeeping gene control GAPDH were included in the Advantage cDNA kit^®^. Control primers amplifying a fragment of the control plasmid included in the Advantage cDNA kit^® ^were used to ensure optimal PCR conditions. PCR was performed using thermocycler (Biometra Tgradient, Goettingen, Germany) conditions of 94°C for 1 min; 25 cycles of 94°C for 30 sec, 68°C for 4 min; 68°C for 5 min and 15°C soak.

For quantitative PCR, total RNA was extracted from the different cell lines. RNA was then converted to cDNA using the High Capacity cDNA Archive Kit (Applied Biosystems Inc. (ABI), Foster City, CA). qPCR analysis was performed using TaqMan probes for HIF-1α (ABI Catalog number Hs00936366) or HIF-1α^785 ^(ABI Custom Primer Order) as well as β-actin (ABI Catalog number 4326315E). The reactions were performed under conditions specified in the ABI TaqMan Gene Quantitation assay protocol. Data was corrected for efficiency and loading using the Pfaffl method [36]. Data is representative of at least 3 separate experiments.

### DNA constructs

The pLenti-V5-D-TOPO vector was used in gain of function experiments in the SbCl2 radial growth phase human melanoma cells. HIF-1α or HIF-1α785 was cloned into this vector by amplifying these genes using primers specific for both the 5' and 3' ends of HIF-1α. The linearized pLenti-V5-D-TOPO Vector contains 5' GTGG overhangs at one end while the insert is Taq Amplified to contain a 5' CACC overhang at one end. Following amplification, 20 μL of the 50 μL PCR amplification reactions were separated on a 1% agarose gel stained with ethidium bromide to ensure that the correct size amplicon was present. HIF-1α and HIF-1α785 amplicons were purified from the remainder of the PCR reaction using the Zymo DNA Clean & Concentrate Kit^® ^(Zymo Research Inc., Orange, CA). After ligation, plasmids were transformed into Stbl3 competent cells (Invitrogen Corp., Carlesbad, CA) and plated onto agar plates containing 100 μg/mL ampicillin. Plates were incubated at 37°C overnight and then colonies were screened for intact insert in the correct orientation. Plasmids were isolated from positive colonies and analyzed by DNA sequencing to ensure the correct plasmid expression construct. Control plasmid, pLenti-V5-LacZ, was supplied in the ViraPower kit^® ^(Invitrogen Corp., Carlsbad, CA).

### Transient expression studies

SbCl2 cells were transfected at ~80% confluence with either transfection reagent alone (mock), pLenti-LacZ, pLenti-V5-D-TOPO-HIF-1α, or pLenti-V5-D-TOPO-HIF-1α785 using FuGene 6 transfection reagent as per manufacturer's protocol (Roche, Palo Alto, CA). After 48 h protein was extracted and overexpression was confirmed by western blot as described above.

### siRNA inhibition of HIF-1α

WM9 cells seeded into 6 well plates at 2.0 × 10^5 ^were treated 24 h after seeding with either 100 nM HIF-1α siRNA or 100 nM control non-targeting siRNA (Dharmacon, Inc. Lafayette, CO) using the RNAifect^® ^transfection reagent (Qiagen, Inc.) as per the manufacturer's instructions. HIF-1α inhibition was confirmed by western blotting at 48, 72, 96, and 120 h after transfection. There was ~70% - 80% decrease in HIF-1α protein relative to control siRNA treated WM9 cells at each time point. Cell viability assays were performed using WM9 cells transfected with control siRNA or HIF-1α siRNA at 48, 72 and 96 h by the trypan blue exclusion method.

### Matrigel invasion assay

SbCl2 cells overexpressing either HIF-1α, HIF-1α785, or LacZ for 24 h, or WM9 cells transfected with Control siRNA or HIF-1α siRNA for 48 h were seeded into 6-well Matrigel (+) chambers, and, as a control, 6-well Matrigel (-) chambers (BD Biosciences) at 7.0 × 10^4 ^cells per well. At 24 hours post-seeding, the Matrigel was removed from the chambers using a cotton-tipped applicator. After all the Matrigel on the inner part of the chambers was removed, invading cells were fixed with 80% methanol for 5 minutes and then stained with 0.5% crystal violet for 5 minutes. After staining, the cells/chambers were extensively washed with distilled water. Once excess stain was removed, cells were manually counted using a grid system covering the entire lower surface of the chamber.

### Anchorage-independent growth assay

CytoSelect 96 well Cell Transformation Assay^® ^(Cell Biolabs, Inc.) was used to determine anchorage-independent growth of SbCl2 cells overexpressing either HIF-1α, HIF-1α785, or LacZ and WM9 cells transfected with Control siRNA or HIF-1α siRNA. Cells were seeded at a density of 1.0 × 10^4 ^into a 0.4% agar layer poured over a 0.6% agar layer in wells of a 96 well plate and incubated for 4-5 days as per manufacturer's instructions. Wells lacking cells served as a fluorescent blank control. Agar layers were solubilized, cells were lysed, and nucleic acid content stained with CyQuant dye. The amount of Cyquant dye in each well was determined using a fluorescent plate detector (Molecular Devices' Spectra Max GEMINI EM Microplate spectrofluorometer; Biocompare Inc., CA) at 485/520 nm. Anchorage-independent growth of WM9 cells transfected with Control siRNA or HIF-1α siRNA for 5 days was also confirmed by microscopic examination (20×).

### ERK inhibition

WM9 cells were seeded into 10 cm dishes at a density of 5.0 × 10^5 ^and the next day were treated with either 30 or 10 μmol/L U0126 (Promega, Madison, WI), a MEK1/2-specific inhibitor to block ERK1/2 activation or vehicle (DMSO). ERK inhibition was verified by western blotting at 24, 48 and 72 h using both total and phospho-specific ERK antibodies.

WM9 cells seeded into 6 well plates at 2.0 × 10^5 ^were treated 24 h after seeding with either 100 nM MEK1 and MEK2 siRNA or 100 nM control non-targeting siRNA (Dharmacon, Inc. Lafayette, CO) using the RNAifect^® ^transfection reagent (Qiagen, Inc.) as per the manufacturer's instructions. Both MEK1 and MEK2 inhibition was confirmed by western blotting at 72 h after transfection. There was ~80 - 90% decrease in MEK1 and MEK2 protein relative to control siRNA treated WM9 cells at each time point.

### Statistics

Statistical analysis of the data was performed using the student paired *t *test or ANOVA as appropriate. The statistical test used for each data set is stated in the figure legends; *p *< 0.05 was considered to be significant. Error bars in all figures represent SEM.

## Competing interests

The authors declare that they have no competing interests.

## Authors' contributions

All authors significantly contributed to the design of the study, data and manuscript drafts. SSJ and CNM contributed equally in carrying out the experiments reported in this study. All authors have read and approved the final manuscript.
